# Commentary: Managing Virtual Hybrid Psychiatrist-Patient Relationships in a Digital World

**DOI:** 10.3389/fpubh.2021.664778

**Published:** 2021-04-13

**Authors:** Robert M. Lundin, David B. Menkes

**Affiliations:** ^1^Adult Mental Health and Addiction Services, Waikato District Health Board, Hamilton, New Zealand; ^2^Waikato Clinical Campus, University of Auckland, Hamilton, New Zealand

**Keywords:** virtual reality, governance, augmented reality, side effects, technology

## Introduction

In his recent JAMA Psychiatry article “Managing Virtual Hybrid Psychiatrist-Patient Relationships in a Digital World,” Shore (1) makes a convincing case for psychiatrists to be familiar with developing technologies as they affect both the doctor-patient relationship and clinical outcomes. His scope usefully identifies administrative, operational, and clinical domains relevant to the use of various technologies, including email, text message, videoconferencing, web-based patient portals, and social networks.

Conspicuously absent from Shore's argument is any consideration of research governance vital to the ethical development and application of these new technologies. He also neglects to mention another clinically promising technology; virtual reality (VR) has been studied in several psychiatric conditions (2) and is distinct in that it places patients completely within a digital, multi-modality, three-dimensional space, and enables direct interaction with that virtual environment. Clinicians need to know that VR is primarily accessed through head-mounted displays and that patient movements within the virtual space can measured by external or internal device sensors; hand-held controllers allow direct manipulation of the virtual environment which can also be measured and analyzed to provide data relevant to treatment optimization (2).

## Virtual Reality in Psychiatry

The more recent availability of consumer-focused VR-devices has led to a flurry of interest and research. In psychiatry this research has thus far primarily focused on virtual versions of established therapies such as cognitive behavioral therapy (CBT) and exposure therapy (ET). These have been usefully applied to specific phobias (3) and eating disorders (4) and, more recently, trialed in schizophrenia (5). VR could also provide students and clinicians of various disciplines the opportunity to experience psychiatric patients' pathological symptoms, such as auditory and visual hallucinations. Beyond psychiatry there are important use-cases ranging from clinical use in pain detection (6) to enhanced training of various healthcare professionals (7).

Extending the framework proposed by Shore, we suggest that the following considerations should apply to the developing use of VR in psychiatry. Administrative concerns include the licensing of platforms and software, and how data are collected, stored, and analyzed. Operational aspects are often more complicated; significant resources and technical expertise required to set up and effectively apply the technology, and to troubleshoot problems. Clinical evidence of VR's usefulness is accumulating with, for example, VR-exposure therapy (VR-ET) producing comparable results to conventional ET; an important advantage of VR is that it provides both therapists and patients greater control in the design and application of therapeutic environments (3).

In addition to the above suggested expansion of Shore's administrative domain (1), and considering the key role research governance plays in the ethical development and application of digital technology, we propose this be regarded as a separate domain. Furthermore, trials conducted for several digital technologies are often tested on non-clinical populations and the literature therefore contains many gaps regarding effects on, for example, clinical depression, and anxiety (2). While this might be due, in part, to ethical challenges associated with testing on severe mental disorders, a focus on clinical populations will be essential to determine the therapeutic place of these technologies. It would also be important to ensure inclusion of a range of disorders and demographics in the research; otherwise clinical advances may be difficult to realize, as has occurred with the difficulties in identifying and treating female patients with autism spectrum disorder as a result of criteria being developed around male patients (8). It is therefore important that clear development, testing and reporting guidelines are developed for VR and other digital technologies used in mental health and early work by expert groups has begun to provide such guidance (9).

The current reliance on proprietary software and platforms and consequent lack of open source alternatives in VR research is an important governance limitation that constrains progress in the development and application of this technology. As with other developing technologies, it is important to consider potential conflicts of interest in the promotion and use of proprietary software and hardware. Finally, as with other emerging therapeutic modalities, there is limited knowledge of the potential adverse effects associated with immersive VR interventions for mental health, and those designing clinical trials should be alert to a range of possible outcomes and considering this when reporting results. This is particularly important when considering that psychological interventions have a particularly poor track record for reporting adverse events (10).

## Discussion

New technologies such as VR may offer a further advantage in light of current concerns about the risk of infection from face-to-face interactions and provide some relief to healthcare organizations and clinicians that have scrambled during the COVID-19 pandemic to offer virtual consultations (11). Mental health clinicians should learn from this and make sure organizations can seamlessly adapt virtual alternatives when necessary; VR can be of particular advantage due to how readily it can be adapted to automated treatment and data collection and the location where treatment is delivered (12). Ethical research governance also represents an important challenge, particularly as new technologies pose new risks in terms of privacy and confidentiality. On the other hand, new technologies can be exceptionally useful, for example allowing new data sources (such as changes in patterns of mobile phone use and geographical data and activity from smart home sensors) that can detect early warning signs of severe mental disorders. These technologies may also be applicable to forensic mental health, for example helping to monitor behaviors associated with risks of relapse or violence. In our pursuit of these advances we must not forget the complicated history of ethics in psychiatry. The advent of new technologies means that sound, ethical research governance will be more important than ever.

## Author Contributions

RL and DM conceptualized the article, and contributed to editing and proofreading. RL wrote the first draft. All authors contributed to the article and approved the submitted version.

## Conflict of Interest

The authors declare that the research was conducted in the absence of any commercial or financial relationships that could be construed as a potential conflict of interest.
